# Prevalence and Factors Influencing Trichotillomania Among Healthcare Workers and Students in Saudi Arabia: A Cross-Sectional Analytical Study

**DOI:** 10.7759/cureus.51128

**Published:** 2023-12-26

**Authors:** Sadeem D Alonazi, Alanoud W AlHnake, Faisal S Alahmari, Eman Abahussain, Abdullah H Alkahtani, Khalid A Alharbi, Ahmed Alasiri

**Affiliations:** 1 Medicine, Imam Mohammad Ibn Saud Islamic University, Riyadh, SAU; 2 College of Medicine, Imam Mohammad Ibn Saud Islamic University, Riyadh, SAU; 3 Neuroscience, Imam Mohammad Ibn Saud Islamic University, Riyadh, SAU; 4 Medicine and Surgery, Imam Mohammad Ibn Saud Islamic University, Riyadh, SAU; 5 Psychiatry, Imam Mohammad Ibn Saud Islamic University, Riyadh, SAU

**Keywords:** prevalence, medical students, medical workers, psychiatric illness, trichotillomania

## Abstract

Background: Trichotillomania (TTM) is a psychiatric disorder characterized by repetitive pulling out of one’s own hair, in which the scalp, brows, and eyelids are the most common sites. This study aimed to measure the prevalence of TTM among healthcare workers and students and to determine its association with psychiatric disorders.

Methodology: This cross-sectional study was conducted among healthcare workers and students in Saudi Arabia. Data were collected through an online self-administered questionnaire. The questionnaire consisted of sociodemographic characteristics, the Massachusetts General Hospital (MGH) Hairpulling Scale to measure TTM, and the Depression and Anxiety Stress Scale (DASS-21) to measure the psychiatric disorders of the participants. A convenience sampling technique was implemented. The sample size was calculated to be 385.

Results: Of the total 460 participants, 62% (n = 285) were students, 55% were females and 61.7% (n = 284) were aged between 18 and 24 years. The most commonly associated chronic disease was diabetes (n = 34, 7.4%), followed by asthma (n = 30, 6.5%). The prevalence of TTM was 4.8% (n = 22), which was higher among medical students (n = 15, 5.3%) as compared to medical workers (n = 7, 4%). While taking psychiatric medication (AOR = 0.197; 95% CI = 0.076-0.508 p = 0.001) was identified as the protective factor for TTM, previous diagnoses of psychiatric illness (AOR = 4.298; 95% CI = 1.759-10.499; p = 0.001), stress (AOR = 4.759; 95% CI = 1.541-14.695; p = 0.007), and depression (AOR = 3.149; 95% CI = 1.190-8.334; p = 0.021) were recognized as independent risk factors of TTM.

Conclusion: Trichotillomania was less common among health workers and students in Saudi Arabia. However, if trichotillomania is present, the disorder was found to be more common among those with associated psychiatric illnesses, including those who were anxious and depressed. Hence, further research is required to validate the impact of psychiatric conditions on the prevalence of TTM in the study region.

## Introduction

Trichotillomania (TTM) is a psychiatric disorder that involves repetitive pulling out of one’s hair. Any area of the body where hair grows can be pulled; nevertheless, the scalp, eyebrows, and eyelids are the common sites. It leads to significant distress or impairment in daily life function [1]. The etiology of TTM is multifactorial and affects females more than males; moreover, it is positively associated with other psychiatric disorders such as obsessive-compulsive disorder (OCD), major depressive disorder, and excoriation disorder [2]. Furthermore, TTM is a chronic disease with fluctuations in intensity with time. However, females might experience worsening symptoms secondary to hormonal changes [1].

Internationally, a cross-sectional study was conducted in the United States using a survey among 10,169 adults, aged 18-69 years. It was found that 1.7% of individuals were identified with current TTM (1.8% males and 1.7% females). In addition, 79% of people with TTM had one or more mental health comorbidities [3].

Locally, a recent study was conducted in 2022 among 511 adults living in Jeddah city of Saudi Arabia and aged 18 years and above to assess symptoms of TTM. The results showed that 40% had a history of hairpulling, while <2% were suspected of TTM. Females (2.8% vs. 0.4% in males, P = 0.047) and those with a history of OCD (6.7% vs. 1.5%, P = 0.093) were more likely to have suspected TTM. Hairpulling was more prevalent among single, non-family residents, and unemployed people. The most frequent areas of hairpulling were the face (62.7%), head (55.7%), and legs (15.3%) [4].

To assess the prevalence of TTM and hairpulling, a systematic review and meta-analysis were conducted. According to meta-analysis, the prevalence of TTM was 1.14% (95% CI: 0.66%, 1.96%), whereas the prevalence of any hairpulling behavior was 8.84% (95% CI: 6.33%, 12.20%). Females were shown to have a higher risk of any hairpulling (OR = 2.23, 95% CI: 1.60%, 3.10%; p = 0.0001). This study showed that TTM potentially affects only 1% of people, but general hairpulling practices affect 8% of people, underscoring the serious public health consequences of this understudied illness [5].

Another study was carried out among medical students of Saint Joseph University and found that TTM affected all students equally and not just medical students. Due to the absence, a screening tool for this disease must be taken into consideration. Psychiatric-trained medical students were more knowledgeable about TTM than others [6].

Another cross-sectional study was conducted in Karachi among 210 students attending three different medical colleges using the “Habit Questionnaire.” The study aimed to determine the prevalence of body-focused repetitive behaviors (BFRBs), and the results showed that BFRBs were more common among females, and TTM was the most common disorder with a prevalence of 13.3% [7]. Further, a study was conducted at Wroclaw Medical University in Poland to determine the prevalence of TTM in young adults and its relationship with OCD and anxiety. It was found that only five participants with TTM were diagnosed with lifetime anxiety disorder (P-value = 0.01), and individuals with TTM were not diagnosed with OCD [8].

Until now, there is only one local study that illustrates the prevalence of TTM among the general population in Jeddah, Saudi Arabia [4]. However, no single study illustrated the prevalence among healthcare workers and students. This study's main objective aimed to assess the prevalence of TTM and address factors influencing it among healthcare workers and students.

## Materials and methods

Study design and study setting

This is an analytical cross-sectional study conducted using a convenient sampling technique from January 2023 to September 2023 through a self-administered online questionnaire among Saudi and non-Saudi healthcare workers and students studying medicine and related fields in five regions (North, South, East, West, and Central) of Saudi Arabia. The questionnaire was distributed through various social media platforms through WhatsApp, Telegram, and other applications as it adhered to the tenets of the Declaration of Helsinki 2013. The study objectives were clearly stated and explained to the participants, and consent was obtained upon this.

Study population

The study population included healthcare workers and students studying medicine and related fields (nursing, pharmacology, applied medical science, dentistry, and others) aged 18 or above who attended government and private medical colleges and hospitals in Saudi Arabia.

Inclusion and exclusion criteria

The study included healthcare workers and students who are studying medicine and related fields (nursing, pharmacology, applied medical science, dentistry, and others) aged 18 years or above residing in Saudi Arabia. Those who were not healthcare workers, students in fields other than medicine or related fields, and who were aged less than 18 years were excluded.

Sample size

We calculated the sample size based on the following formula:

Sample size = Z^2^p(1-p)/c^2^

where Z = Z value, which is also known as confidence level = 1.96 for 95%; p = percentage picking a choice = 0.5; c = confidence interval = ±5% = 0.05. 

The minimum sample size needed to achieve a precision of ±5% with a 95% confidence interval of 385.

Study tool

The questionnaire was divided into three categories: The first part investigated the participants’ demographic data and obtained consent from them by asking them "Do you agree to participate in the study?" (yes/no), and those who answered "no" did not complete the questionnaire; the second part assessed TTM among the participants through the Massachusetts General Hospital Hairpulling Scale (MGH-HPS), and the third part assessed the depression, anxiety, and stress levels through Depression, Anxiety and Stress Scale-21 (DASS-21) items.

The assessment of TTM was measured using the MGH-HPS, developed by Keuthen et al. [9]. The MGH-HPS is a seven-item measure of hairpulling symptoms. Each item is scored on a five-point scale from 0 (no symptoms) to 4 (severe symptoms). MGH scores range from 0 to 28 points. The higher the score, the higher the severity of hairpulling. A cutoff point of 17 or higher is considered to be essential for the diagnosis [4].

Likewise, anxiety, depression, and stress levels were assessed using the DASS-21 developed by Lovibond and Lovibond [10]. Each mental disorder domain has 14 items, and each item has four choices: “never” coded with 0, "sometimes" coded with 1, "often" coded with 2, and “almost always” coded with 3. All items (14 each) were summed up to obtain the total score, with a maximum of 42 points. The severity was classified into five levels depending on the score. For stress, normal (0-10 points), mild (11-18 points), moderate (19-29 points), severe (27-34 points), and extremely severe (35-42). For anxiety, normal (0-6 points), mild (7-9 points), moderate (10-14 points), severe (15-19 points), and extremely severe (20-42). Finally, for depression, normal (0-9 points), mild (10-12 points), moderate (13-20 points), severe (21-27 points), and extremely severe (28-42) [10].

Statistical analysis

The data were analyzed using the Statistical Package for the Social Sciences (SPSS) software, version 26 (IBM Corp., Armonk, NY). Descriptive statistics were given as numbers and percentages (%) for all categorical variables, while continuous variables were calculated and tabulated as mean and standard deviation. The comparison between medical field workers and students according to mental disorders and TTM was conducted using the Chi-square and Mann-Whitney U tests. The statistical enumeration extends to normality tests that were performed using the Shapiro-Wilk test and the Kolmogorov-Smirnov test, in addition to delving into the relationship of sociodemographic characteristics and mental disorders with TTM and quantifying it with the Chi-square test. Significant results were then tested in a multivariate regression analysis to determine the significant independent risk factors of TTM with corresponding odds ratios and 95% confidence interval. Values were considered significant with a p-value of less than 0.05.

Ethical considerations

The study was approved by Imam Mohammad Ibn Saud Islamic University IRB via reference number 457/2023 dated 20/5/2023.

## Results

A total of 460 participants filled out the survey. The most common age group was 18-24 years (n = 284, 61.7%), with more than half being females (n = 253, 55%). Participants who lived in the central region constituted 48.9% (n = 225). Most of the participants were single (n = 396, 86.1%) and without children (n = 411, 89.3%). Participants who had four to six family members living in the same household constituted 37.8% (n = 174). Approximately 56.1% (n = 258) indicated high family support. In addition, 86.1% (n = 396) had no previous psychiatric illness (Table 1).

**Table 1:** Sociodemographic characteristics of the medical field workers and students (n = 460) SSRIs: Selective serotonin reuptake inhibitors; CBT: Cognitive behavioral therapy. The data has been represented as N and %.

Study data	N (%)
Age group	
18–24 years	284 (61.7)
25–29 years	139 (30.2)
30–34 years	17 (03.7)
35–39 years	13 (02.8)
≥40 years	07 (01.5)
Gender	
Male	207 (45.0)
Female	253 (55.0)
Residence	
Eastern Region	47 (10.2)
Western Region	47 (10.2)
Central Region	225 (48.9)
Southern Region	76 (16.5)
Northern Region	65 (14.1)
Marital status	
Single	396 (86.1)
Married	43 (09.3)
Divorced	12 (02.6)
Widowed	09 (02.0)
Number of children	
None	411 (89.3)
1–2	31 (06.7)
3–5	12 (02.6)
>5	06 (01.3)
Number of family members living in the same household	
None	46 (10.0)
1–3	87 (18.9)
4–6	174 (37.8)
7–9	116 (25.2)
>9	37 (08.0)
Family support (out of 10)	
Low (1–4)	96 (20.9)
Average (5–7)	106 (23.0)
High (8–10)	258 (56.1)
If you have been diagnosed with any of the previous psychiatric illnesses, are you currently in treatment?	
None	396 (86.1)
SSRIs	53 (11.5)
CBT	11 (02.4)

Participants with bachelor’s degrees constituted 32% (n = 147) of the sample. The majority of the participants (n = 355, 77.2%) were working/studying in a medicine specialty. Among students (n = 285), 16.1% were in the internship levels, and 29.5% (n = 84) had a 4.75 and above GPA level. Among workers (n = 175), 49.1% had at least less than a year of working experience (Table 2).

**Table 2:** Educational profile of the medical health workers and students (n = 460) The data has been represented as N and %.

Study data	N (%)
Level of education	
Student	285 (62.0)
Bachelor	147 (32.0)
Master degree	24 (05.2)
PhD	04 (0.90)
What type of medical field are you studying/working currently?	
Medicine	355 (77.2)
Nursing	24 (05.2)
Pharmacology	21 (04.6)
Applied medical science	24 (05.2)
Dentistry	24 (05.2)
Others	12 (02.6)
If you are a student, which year of the program are you currently studying? (n = 285)	
Preparatory year	08 (02.8)
1st year	29 (10.2)
2nd year	69 (24.2)
3rd year	53 (18.6)
4th year	41 (14.4)
5th year	39 (13.7)
Internship	46 (16.1)
Your GPA level currently (out of 5) (n = 285)	
<3	21 (07.4)
3.0–3.49	23 (08.1)
3.49–3.99	46 (16.1)
4.0–4.49	56 (19.6)
4.49–4.74	55 (19.3)
4.75 and above	84 (29.5)
If you are a worker, how long have you been working in the medical field? (n = 175)	
<1 years	86 (49.1)
1–4 years	52 (29.7)
5–9 years	15 (08.6)
10–14 years	13 (07.4)
≥15 years	09 (05.1)

**Figure 1:**
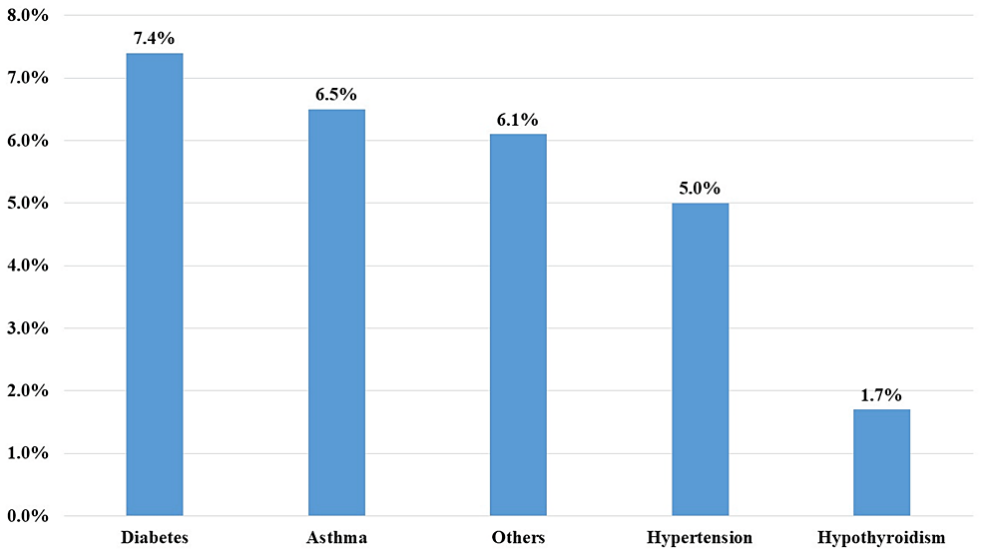
Associated chronic diseases among the participants Other diseases include arthritis, inflammatory bowel diseases, stroke, obesity, cardiovascular diseases, etc.

The most commonly associated chronic disease was diabetes (n = 34, 7.4%), followed by asthma (n = 30, 6.5%) (Figure 1).

**Figure 2:**
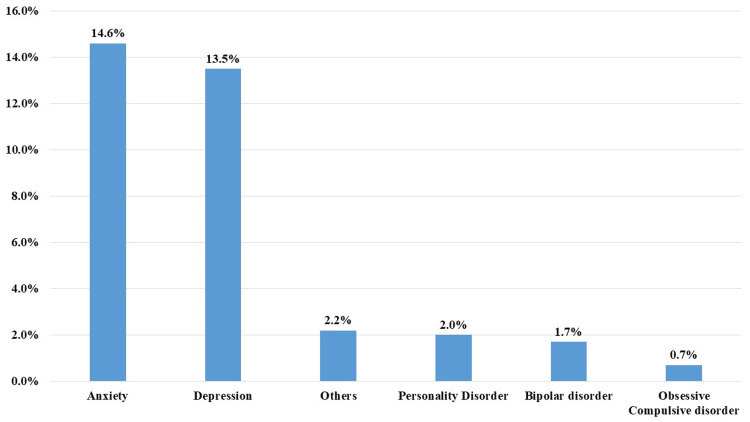
Associated psychiatric diseases among the participants

The most commonly associated psychiatric diseases were anxiety (n = 67, 14.6%) and depression (n = 62, 13.5%) (Figure 2).

It was observed that the mean scores for stress, anxiety, and depression were 11.5 ± 10.2, 10.0 ± 9.52, and 10.1 ± 10.5, respectively. According to the scores of DASS-21, 48.9% (n = 225) of participants were stressed, 54.6% (n = 251) were anxious, and 45.9% (n = 211) were depressed. Regarding TTM, the overall mean score was 3.43 (SD 5.77), where 4.8% of participants had positive symptoms. Regarding the comparison of the prevalence between medical students and medical workers, it was observed that medical students had a higher mean score in stress (p = 0.001), anxiety (p < 0.001), and depression (p = 0.040). Similarly, it was observed that the prevalence of extremely severe stress (p = 0.009), stress (p < 0.001), extremely severe anxiety (p < 0.001), and anxiety (p = 0.016) was significantly more common among medical students (Table 3).

**Table 3:** Prevalence of mental disorders using DASS-21 and TTM using MGH-HPS among medical health workers and students The data has been represented as both N and % as well as mean ± SD. ^§^ P-value has been calculated using the Chi-square test and the Mann-Whitney test. ** Significant at P < 0.05 level. DASS: Depression and Anxiety Stress Scale; TTM: Trichotillomania; MGH-HPS: Massachusetts General Hospital Hairpulling Scale.

Variables	Overall N (%) (n = 460)	Medical students (n = 285)	Medical workers (n = 175)	P-value^§^
Stress score (mean ± SD)	11.5 ± 10.2	12.6 ± 1.4	9.59 ± 9.67	0.001**
Severity of stress				
Normal (0–10)	235 (51.1%)	128 (44.9%)	107 (61.1%)	0.009**
Mild (11–18)	126 (27.4%)	86 (30.2%)	40 (22.9%)	
Moderate (19–26)	56 (12.2%)	42 (14.7%)	14 (08.0%)	
Severe (27–34)	32 (07.0%)	20 (07.0%)	12 (06.9%)	
Extremely severe (35–42)	11 (02.4%)	09 (03.2%)	02 (01.1%)	
Level of stress				
Stressed (11–42)	225 (48.9%)	157 (55.1%)	68 (38.9%)	0.001**
Not stressed (0–10)	235 (51.1%)	128 (44.9%)	107 (61.1%)	
Anxiety score (mean ± SD)	10.0 ± 9.52	11.4 ± 9.87	7.87 ± 8.50	<0.001**
Severity of anxiety				
Normal (0–6)	209 (45.4%)	117 (41.1%)	92 (52.6%)	<0.001**
Mild (7–9)	30 (06.5%)	14 (04.9%)	16 (09.1%)	
Moderate (10–14)	84 (18.3%)	48 (16.8%)	36 (20.6%)	
Severe (15–19)	53 (11.5%)	40 (14.0%)	13 (07.4%)	
Extremely severe (20–42)	84 (18.3%)	66 (23.2%)	18 (10.3%)	
Level of anxiety				
Anxious (7–42)	251 (54.6%)	168 (58.9%)	83 (47.4%)	0.016**
Not anxious (0–6)	209 (45.4%)	117 (41.1%)	92 (52.6%)	
Depression score (mean ± SD)	10.1 ± 10.5	10.8 ± 10.7	9.03 ± 10.1	0.040**
Severity of depression				
Normal (0–9)	249 (54.1%)	148 (51.9%)	101 (57.7%)	0.523
Mild (10–12)	65 (14.1%)	40 (14.0%)	25 (14.3%)	
Moderate (13–20)	70 (15.2%)	47 (16.5%)	23 (13.1%)	
Severe (21–27)	34 (07.4%)	20 (07.0%)	14 (08.0%)	
Extremely severe (28–42)	42 (09.1%)	30 (10.5%)	12 (06.9%)	
Level of depression				
Depressed (10–42)	211 (45.9%)	137 (48.1%)	74 (42.3%)	0.227
Not depressed (0–9)	249 (54.1%)	148 (51.9%)	101 (57.7%)	
TTM score (mean ± SD)	3.43 ± 5.77	3.66 ± 6.02	3.05 ± 5.33	0.322
Prevalence of TTM				
Positive (≥17)	22 (04.8%)	15 (05.3%)	07 (04.0%)	0.538
Negative (<17)	438 (95.2%)	270 (94.7%)	168 (96.0%)	

While measuring the relationship between TTM according to the sociodemographic characteristics and previous diagnosis of mental disorder, it was found that the prevalence of participants who were positive for the symptoms of TTM was significantly more common among those who were previously diagnosed with psychiatric illness (p < 0.001), those who were not taking medication for psychiatric illness (p < 0.001), those who were stressed (p = 0.002), those who were anxious (p = 0.028), and those who were depressed (p = 0.010) (Table 4).

**Table 4:** Relationship between TTM among the sociodemographic characteristics and mental disorders of the medical field workers and students (n = 460) The data has been represented as N and %. ^§^ P-value has been calculated using the Chi-square test. ** Significant at p < 0.05 level. TTM: Trichotillomania.

Factor	Trichotillomania		P-value^§^
	Positive N (%) (n = 22)	Negative N (%) (n = 438)	
Age group			
<25 years	15 (68.2%)	269 (61.4%)	0.521
≥25 years	07 (31.8%)	169 (38.6%)	
Gender			
Male	07 (31.8%)	200 (45.7%)	0.203
Female	15 (68.2%)	238 (54.3%)	
Residence			
Outside central region	09 (40.9%)	226 (51.6%)	0.328
Inside central region	13 (59.1%)	212 (48.4%)	
Marital status			
Never been married	19 (86.4%)	377 (86.1%)	0.969
Been married	03 (13.6%)	61 (13.9%)	
Having children			
Yes	04 (18.2%)	45 (10.3%)	0.241
No	18 (81.8%)	393 (89.7%)	
Family support (out of 10)			
Low (1–4)	06 (27.3%)	90 (20.5%)	0.704
Average (5–7)	04 (18.2%)	102 (23.3%)	
High (8–10)	12 (54.5%)	246 (56.2%)	
Type of field currently studying or working			
Medical field	17 (77.3%)	338 (77.2%)	0.991
Non-medical field	05 (22.7%)	100 (22.8%)	
Associated chronic disease			
Yes	06 (27.3%)	95 (21.7%)	0.537
No	16 (72.7%)	343 (78.3%)	
Associated psychiatric illness			
Yes	12 (54.5%)	95 (21.7%)	<0.001 **
No	10 (45.5%)	343 (78.3%)	
Taking medication for psychiatric illness			
Yes	09 (40.9%)	55 (12.6%)	<0.001 **
No	13 (59.1%)	383 (87.4%)	
Level of stress			
Stressed	18 (81.8%)	207 (47.3%)	0.002 **
Not stressed	04 (18.2%)	231 (52.7%)	
Level of anxiety			
Anxious	17 (77.3%)	234 (53.4%)	0.028 **
Not anxious	05 (22.7%)	204 (46.6%)	
Level of depression			
Depressed	16 (72.7%)	195 (44.5%)	0.010 **
Not depressed	06 (27.3%)	243 (55.5%)	

A multivariate regression estimate revealed that associated psychiatric illness, stress, and depression were identified as the significant independent risk factors for positive TTM, whereas taking psychiatric medication was identified as a significant independent preventive factor for TTM. This further suggested that participants with psychiatric illness were 4.3 times more likely to have a symptom of TTM than those who were not diagnosed with mental illness (AOR = 4.298; 95% CI = 1.759-10.499; p = 0.001). Compared to participants who were not stressed, participants who were stressed were predicted to have an increased risk of TTM by at least 4.8 times (AOR = 4.759; 95% CI = 1.541-14.695; p = 0.007). Compared to participants who were not depressed, depressed participants were predicted to have an increased risk of TTM by at least 3.1-fold (AOR = 3.149; 95% CI = 1.190-8.334; p = 0.021). In contrast, participants who took psychiatric medication were predicted to have decreased risk of TTM by almost 80% (AOR = 0.197; 95% CI = 0.076-0.508; p = 0.001) (Table 5).

**Table 5:** Multivariate regression analysis to determine the significant independent risk factors for trichotillomania (n = 460) Adjusted with age, gender, marital status, and chronic disease. ** Significant at p < 0.05 level. AOR: Adjusted Odds ratio; CI: Confidence interval.

Factors	AOR	95% CI	P-value
Associated psychiatric illness			
Yes	4.298	1.759–10.499	0.001**
No	Ref		
Taking medication for psychiatric illness			
Yes	0.197	0.076–0.508	0.001**
No	Ref		
Level of stress			
Stressed	4.759	1.541–14.695	0.007**
Not stressed	Ref		
Level of anxiety			
Anxious	2.713	0.955–7.702	0.061
Not Anxious	Ref		
Level of depression			
Depressed	3.149	1.190–8.334	0.021**
Not depressed	Ref		

## Discussion

This study was carried out to determine the prevalence and the influencing factors of trichotillomania among medical field workers and students. The prevalence of TTM in this study was 4.8% (n = 22), with TTM being more common in medical students (n = 15, 5.3%) than medical field workers (n = 7, 4.0%), but without significant difference (p = 0.322). These findings can be compared with the study of Mikhael et al. who compared the rates of TTM between medical students and the general population, and it was observed that the rate of TTM among the general population (3.3%) was higher than that among medical students (0.9%). The overall prevalence of TTM among the Lebanese population was 2.2% [6].

However, Subki et al. [4] found that the overall prevalence of TTM among the Arab Middle East population was only 1.8% lower than the current reports. Contradicting these reports, Siddique et al. [7] documented a higher prevalence of TMM among students at 13.3%. According to the systematic review and meta-analysis conducted by Thomson et al. [5], the pool prevalence of TTM was 8.84%.

Participants who were diagnosed with psychiatric illness were more likely to exhibit symptoms of TTM. According to the current multivariate regression estimates, the risk of TTM could increase up to 4.2-fold higher compared to those without mental illness. In South Africa [11], both OCD and TTM were likely to get worse during menstruation. However, OCD patients had a greater lifetime disability than TTM patients.

In the United States [12], the strength of the TTM association was higher in kleptomania, pyromania, OCD, skin-picking disorder, bulimia nervosa, and pathological nail biting. Another study documented in the United States [13] cited that individuals with hairpulling disorder had concurrent psychiatric disease in as much as 80% of the TTM subjects. The presence of psychiatric illness among TTM patients is proven in the literature, and the current study provides evidence supporting this claim. Thus, psychological screening among this group of patients is beneficial for the therapeutic outcome.

In the current univariate analysis, stress, anxiety, and depression were found to be the significant risk factors for TTM. However, in the multivariate regression model adjusted with confounders, only stress and depression remained significant and were determined as the significant independent risk factors for TTM. This was comparable to the study by Özten et al. [14], where both depression and traumatic stress were higher in the TTM group than in the healthy group. Also, compared to health subjects, participants with TTM and skin-picking were more associated with a greater frequency of traumatic and adverse events during their childhood.

In a study published by Grant et al. [15], it was reported that TTM participants with associated anxiety demonstrated severe hairpulling symptoms. Also, they were more likely to exhibit depression when having first-degree relatives with OCD. Other studies reported direct associations between major depressive disorders and other body-focused repetitive behavior disorders [16,17].

In a previous cohort study conducted among the Arab population [1], it was revealed that females were more likely to have TTM and had a history of OCD. Further, TTM was also frequent in unmarried participants, unemployed participants, and those who were not living with their families. Contradicting these reports, Grant et al. [16] disclosed that TTM males were significantly more likely to pull hair from their face, torso, and arms and had an increasing risk of substance use disorder, while TTM females were more likely to be younger and unmarried.

On the contrary, another study by Ganti [3] found insignificant relations between TTM in terms of age, gender, educational level, household income, and racial-ethnic group (p > 0.05). The current results almost mirror this as insignificant associations between TTM among age group, gender, residence region, marital status, having children, family support, and associated chronic disease were found (all p > 0.05).

It is important to note that taking psychiatric medication could decrease the risk of TTM by almost 80%, which was identified as a preventive factor for TTM. In a study conducted in the United States [17], results indicated that males were less likely to seek treatment for TTM, suggesting that sex differences might have to be considered when treating a patient with TTM. The preventive factors for TTM have not been studied well. Hence, more investigations are warranted to determine the protective factor for TTM.

The results of this study were bound to some limitations. The survey used social media platforms to fill out the questionnaire. Hence, participants might not be truthful with their responses to questions. Finally, being a cross-sectional survey, it was prone to bias and did not measure cause and effect. However, this study was able to assess the prevalence of TTM in Saudi Arabia generally and its underlying associations with various influencing factors among healthcare workers and students which would aid in psychological screening and would help policymakers to design interventions that are necessary to prevent the increasing prevalence of mental health conditions.

## Conclusions

Participants who were diagnosed with psychiatric illnesses, such as anxiety and depression, were more likely to exhibit symptoms of trichotillomania when compared to the rest of the population. Furthermore, taking psychiatric medication could prevent suffering from the symptoms of TTM. Psychological screening and interventions are necessary to prevent the increasing prevalence of mental health conditions, which might help in clipping the burden of TTM.

## Appendices

**Table 6:** The Massachusetts General Hospital (MGH) Hairpulling Scale

The Massachusetts General Hospital (MGH) Hairpulling Scale Subject; Number: and Date:gf
Instructions: For each question, pick the one statement in that group, which best describes your behaviors and/or feelings over the past week. If you have been having ups and downs, try to estimate an average for the past week. Be sure to read all the statements in each group before making your choice.
For the next three questions, rate only the urge to pull your hair.
Frequency of urges: On an average day, how often did you feel the urge to pull your hair?
0: This week I felt no urges to pull my hair.
1: This week I felt an occasional urge to pull my hair.
2: This week I felt an urge to pull my hair often.
3: This week I felt an urge to pull my hair very often.
4: This week I felt near-constant urges to pull my hair.
Intensity of urges: On an average day, how intense or "strong" were the urges to pull your hair?
0: This week I did not feel any urge to pull my hair.
1: This week I felt mild urges to pull my hair.
2: This week I felt moderate urges to pull my hair.
3: This week I felt severe urges to pull my hair.
4:This week I felt extreme urges to pull my hair.
Ability to control the urges: On an average day, how much control do you have over the urges to pull your hair?
0: This week I could always control the urges, or I did not feel any urges to pull my hair.
1: This week I was able to distract myself from the urge to pull my hair most of the time.
2: This week I was able to distract myself from the urges to pull my hair some of the time.
3: This week I was able to distract myself from the urge to pull my hair rarely.
4: This week I was never able to distract myself from the urges to pull my hair.
For the next three questions, rate only the actual hairpulling.
Frequency of hairpulling: On an average day, how often did you actually pull your hair?
0: This week I did not pull my hair.
1: This week I pulled my hair occasionally.
2: This week I pulled my hair often.
3: This week I pulled my hair very often.
4: This week I pulled my hair so often, it felt like I was always doing it.
Attempts to resist hairpulling: On an average day, how often did you make an attempt to stop yourself from actually pulling your hair?
0: This week I felt no urges to pull my hair.
1: This week I tried to resist the urge to pull my hair almost all of the time.
2: This week I tried to resist the urge to pull my hair some of the time.
3: This week I tried to resist the urge to pull my hair rarely.
4:This week I never tried to resist the urge to pull my hair.
Control over hairpulling: On an average day, how often were you successful at actually stopping yourself from pulling your hair?
0: This week I did not pull my hair.
1: This week I was able to resist pulling my hair almost all of the time.
2: This week I was able to resist pulling my hair most of the time.
3: This week I was able to resist pulling my hair some of the time.
4: This week I was rarely able to resist pulling my hair.
For the last question, rate the consequences of your hairpulling.
Associated distress: Hairpulling can make some people feel moody, "on edge," or sad. During the past week, how uncomfortable did your hairpulling make you feel?
0: This week I did not feel uncomfortable about my hairpulling.
1: This week I felt vaguely uncomfortable about my hairpulling.
2: This week I felt noticeably uncomfortable about my hairpulling.
3: This week I felt significantly uncomfortable about my hairpulling.
4: This week I felt intensely uncomfortable about my hairpulling.

**Table 7:** DASS 21 DASS: Depression and Anxiety Stress Scale.

1 (s)	I found it hard to wind down	0	1	2	3
2 (a)	I was aware of the dryness in my mouth.	0	1	2	3
3 (d)	I couldn’t seem to experience any positive feelings at all.	0	1	2	3
4 (a)	I experienced breathing difficulty (e.g., excessively rapid breathing, breathlessness in the absence of physical exertion).	0	1	2	3
5 (d)	I found it difficult to work up the initiative to do things.	0	1	2	3
6 (s)	I tended to overreact to situations.	0	1	2	3
7 (a)	I experienced trembling (e.g., in the hands).	0	1	2	3
8 (s)	I felt that I was using a lot of nervous energy.	0	1	2	3
9 (a)	I was worried about situations in which I might panic and make a fool of myself.	0	1	2	3
10 (d)	I felt that I had nothing to look forward to.	0	1	2	3
11 (s)	I found myself getting agitated.	0	1	2	3
12 (s)	I found it difficult to relax.	0	1	2	3
13 (d)	I felt downhearted and blue.	0	1	2	3
14 (s)	I was intolerant of anything that kept me from getting on with what I was doing.	0	1	2	3
15 (a)	I felt I was close to panic.	0	1	2	3
16 (d)	I was unable to become enthusiastic about anything.	0	1	2	3
17 (d)	I felt I wasn’t worth much as a person.	0	1	2	3
18 (s)	I felt that I was rather touchy.	0	1	2	3
19 (a)	I was aware of the action of my heart in the absence of physical exertion (e.g., sense of heart rate increase, heart missing a beat).	0	1	2	3
20 (a)	I felt scared without any good reason.	0	1	2	3
21 (d)	I felt that life was meaningless.	0	1	2	3
